# An update to the distribution of invasive *Ctenolepisma longicaudatum* Escherich in northern Europe, with an overview of other records of Estonian synanthropic bristletails (Insecta: Zygentoma)

**DOI:** 10.3897/BDJ.9.e61848

**Published:** 2021-01-27

**Authors:** Kaarel Sammet, Mati Martin, Tõnu Kesküla, Olavi Kurina

**Affiliations:** 1 Estonian University of Life Sciences, Institute of Agricultural and Environmental Sciences, Tartu, Estonia Estonian University of Life Sciences, Institute of Agricultural and Environmental Sciences Tartu Estonia; 2 Natural History Museum, University of Tartu, Tartu, Estonia Natural History Museum, University of Tartu Tartu Estonia

**Keywords:** invasive species, *Ctenolepisma longicaudatum*, northern Europe

## Abstract

**Background:**

Previously, two species of Zygentoma have been reported as synanthropic in Estonia (*Lepisma saccharinum* Linnaeus, 1758 and *Thermobia domestica* (Packard, 1873)). *Ctenolepisma longicaudatum* Escherich, 1905 is an invasive species that is currently expanding its range in Europe, but had no published records from the northern Baltic Region.

**New information:**

*Ctenolepisma longicaudatum* was first found in Estonia in 2018. It has currently several established populations in public buildings in Tartu and Tallinn, but has not been found in private households, nor in other places in Estonia. A brief overview of its invasion history in northern Europe is given.

## Introduction

Zygentoma Börner, 1904 is a small order (with over 500 described species; Zhang 2013), of primitive insects. They are more common in warmer climates, but some species of Zygentoma are synanthropic and distributed worldwide. Two species of Zygentoma – *Lepisma saccharinum* Linnaeus, 1758 (= *Lepisma saccharina*, as widely used prior to the ICZN decision, International Commission on Zoological Nomenclature 2018) and *Thermobia domestica* (Packard, 1873) – have been previously recorded from Estonia (see below), both only indoors in human settlements. *Ctenolepisma longicaudatum* Escherich, 1905 is an invasive synanthropic species that has been rapidly expanding its range in recent years (Goddard et al. 2016, Kulma et al. 2018, Lock 2007, Meineke and Menge 2014, Pape and Wahlstedt 2002, Thomsen et al. 2019) and is considered a pest of paper and stored materials.

*Ctenolepisma longicaudatum* was first found in Estonia in 2018 and has since been detected at multiple locations. Here, we report for the first time these findings and provide an overview of recent knowledge of that and two other inavsive species of Zygentoma in Estonia. In addition, the invasion history of *C. longicaudatum* in northern Europe is summarised.

## Materials and methods

All preserved material of Zygentoma in Estonian natural history collections was examined and a list of earlier literature records was compiled. Specimens were actively searched for in suitable habitats and about 30 volunteers were asked to report sightings and, if possible, collect specimens of larger than usual silverfish (in 2020). Most material is preserved in 80% ethanol and some specimens were mounted on to microscope slides after clearing in 20% potassium hydroxide (KOH). All studied material is deposited in the Entomological Collection of Estonian University of Life Sciences (IZBE) and the private insect collection of Allan Selin.

## Taxon treatments

### Ctenolepisma longicaudatum

Escherich, 1905

C34EE50F-8835-5436-A854-41BC5C1DF20D

#### Materials

- **Type status:** Other material. **Occurrence:** catalogNumber: IZBE0880013; recordedBy: Tőnu Kesküla; preparations: specimen in alcohol; **Taxon:** scientificName: Ctenolepisma longicaudatum; genus: Ctenolepisma; specificEpithet: longicaudatum; **Location:** country: Estonia; locality: Tartu; verbatimLocality: Estonian University of Life Sciences; decimalLatitude: 58.39219; decimalLongitude: 26.69395; coordinateUncertaintyInMeters: 50; **Identification:** identifiedBy: Kaarel Sammet; **Event:** eventDate: 01.X.2018; **Record Level:** type: PhysicalObject; basisOfRecord: PreservedSpecimen
- **Type status:** Other material. **Occurrence:** catalogNumber: IZBE0880010; preparations: specimen in alcohol; **Taxon:** scientificName: Ctenolepisma longicaudatum; genus: Ctenolepisma; specificEpithet: longicaudatum; **Location:** country: Estonia; locality: Tartu; verbatimLocality: National Archives of Estonia; decimalLatitude: 58.36578; decimalLongitude: 26.69236; coordinateUncertaintyInMeters: 50; **Identification:** identifiedBy: Mati Martin; **Event:** eventDate: 05.VI.2019; **Record Level:** type: PhysicalObject; basisOfRecord: PreservedSpecimen
- **Type status:** Other material. **Occurrence:** preparations: specimen in alcohol; **Taxon:** scientificName: Ctenolepisma longicaudatum; genus: Ctenolepisma; specificEpithet: longicaudatum; **Location:** country: Estonia; locality: Tartu; verbatimLocality: National Archives of Estonia; decimalLatitude: 58.36578; decimalLongitude: 26.69236; coordinateUncertaintyInMeters: 50; **Identification:** identifiedBy: Mati Martin, Kaarel Sammet; **Event:** eventDate: 28.V.2020; **Record Level:** type: PhysicalObject; basisOfRecord: PreservedSpecimen
- **Type status:** Other material. **Occurrence:** catalogNumber: IZBE0880023; recordedBy: Tőnu Kesküla; preparations: specimen in alcohol; **Taxon:** scientificName: Ctenolepisma longicaudatum; genus: Ctenolepisma; specificEpithet: longicaudatum; **Location:** country: Estonia; locality: Tartu; verbatimLocality: Estonian University of Life Sciences; decimalLatitude: 58.39219; decimalLongitude: 26.69395; coordinateUncertaintyInMeters: 50; **Identification:** identifiedBy: Kaarel Sammet; **Event:** eventDate: 14.VIII.2020; **Record Level:** type: PhysicalObject; basisOfRecord: PreservedSpecimen
- **Type status:** Other material. **Occurrence:** catalogNumber: IZBE0880015; recordedBy: Tőnu Kesküla; preparations: specimen in alcohol; **Taxon:** scientificName: Ctenolepisma longicaudatum; genus: Ctenolepisma; specificEpithet: longicaudatum; **Location:** country: Estonia; locality: Tartu; verbatimLocality: Estonian University of Life Sciences; decimalLatitude: 58.39219; decimalLongitude: 26.69395; coordinateUncertaintyInMeters: 50; **Identification:** identifiedBy: Kaarel Sammet; **Event:** eventDate: 23.IX.2019; **Record Level:** type: PhysicalObject; basisOfRecord: PreservedSpecimen
- **Type status:** Other material. **Occurrence:** catalogNumber: IZBE0880022; recordedBy: Märt Kruus; preparations: specimen in alcohol; **Taxon:** scientificName: Ctenolepisma longicaudatum; genus: Ctenolepisma; specificEpithet: longicaudatum; **Location:** country: Estonia; locality: Tartu; verbatimLocality: Estonian University of Life Sciences; decimalLatitude: 58.39219; decimalLongitude: 26.69395; coordinateUncertaintyInMeters: 50; **Identification:** identifiedBy: Kaarel Sammet; **Event:** eventDate: 25.VIII.2020; **Record Level:** type: PhysicalObject; basisOfRecord: PreservedSpecimen
- **Type status:** Other material. **Occurrence:** catalogNumber: IZBE0880024; recordedBy: Ülle Jäe; preparations: dried specimen; **Taxon:** scientificName: Ctenolepisma longicaudatum; genus: Ctenolepisma; specificEpithet: longicaudatum; **Location:** country: Estonia; locality: Tartu; verbatimLocality: Estonian National Museum; decimalLatitude: 58.39588; decimalLongitude: 26.7464; coordinateUncertaintyInMeters: 50; **Identification:** identifiedBy: Kaarel Sammet; **Event:** eventDate: 10.VIII.2020; **Record Level:** type: PhysicalObject; basisOfRecord: PreservedSpecimen
- **Type status:** Other material. **Occurrence:** recordedBy: Erika Alexandra Milani; preparations: specimen in alcohol; **Taxon:** scientificName: Ctenolepisma longicaudatum; genus: Ctenolepisma; specificEpithet: longicaudatum; **Location:** country: Estonia; locality: Tartu; verbatimLocality: Tartu, Kvartal supermarket; decimalLatitude: 58.37701; decimalLongitude: 26.72889; coordinateUncertaintyInMeters: 50; **Identification:** identifiedBy: Kaarel Sammet, Olavi Kurina; **Event:** eventDate: 23.X.2020; **Record Level:** type: PhysicalObject; basisOfRecord: PreservedSpecimen
- **Type status:** Other material. **Occurrence:** recordedBy: Kadri Pärtel; preparations: specimen in alcohol; **Taxon:** scientificName: Ctenolepisma longicaudatum; genus: Ctenolepisma; specificEpithet: longicaudatum; **Location:** country: Estonia; locality: Tartu; verbatimLocality: Tartu University, Chemicum (Fungal herbarium); decimalLatitude: 58.36739; decimalLongitude: 26.69281; coordinateUncertaintyInMeters: 50; **Identification:** identifiedBy: Kaarel Sammet, Olavi Kurina; **Event:** eventDate: 09.XI.2020; **Record Level:** type: PhysicalObject; basisOfRecord: PreservedSpecimen
- **Type status:** Other material. **Occurrence:** catalogNumber: IZBE0880032; recordedBy: Ann Aaresild; preparations: dried specimen; **Taxon:** scientificName: Ctenolepisma longicaudatum; genus: Ctenolepisma; specificEpithet: longicaudatum; **Location:** country: Estonia; locality: Tallinn; verbatimLocality: Tallinn, Pirita tee 56 (Estonian History Museum laboratory); decimalLatitude: 59.4525; decimalLongitude: 24.81013; coordinateUncertaintyInMeters: 50; **Identification:** identifiedBy: Kaarel Sammet, Olavi Kurina; **Event:** eventDate: 16.XII.2020; **Record Level:** type: PhysicalObject; basisOfRecord: PreservedSpecimen

#### Notes

**Figure 1.**
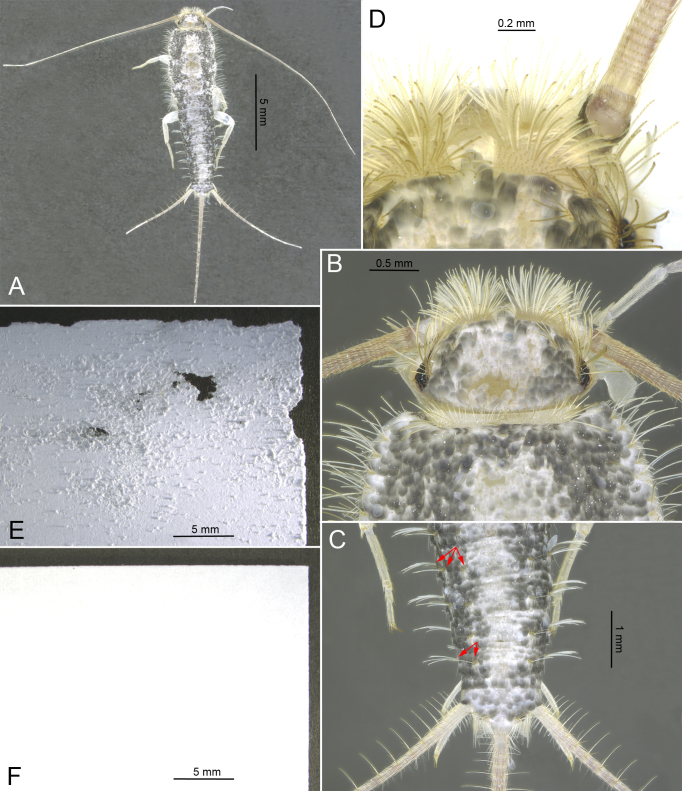
*Ctenolepisma longicaudatum* Escherich, 1905 (A–D) and a comparison of damaged (E) and undamaged (F) paper. **A.** General facies, dorsal view; part of dorsal scales are detached; **B.** Closer view of the head, dorsal view; **C.** Posterior part of the abdomen, dorsal view; red arrows show three bristle-combs on abdominal tergite V and two bristle-combs on abdominal tergite VIII; **D.** Closer view of feathered setae on the head.

First registered in Estonia in 2018 in Tartu (see the Materials and Methods section), now clearly established there (being repeatedly collected or observed in five localities over the period of two years). First found in Tallinn in 2020. No published records. The species is easily distinguished from related synanthropic species by its relatively large size (up to 18 mm in adults), feathered setae, long antennae and caudal filaments, abdominal tergites II – VI with three and tergites VII-IX with two pairs of bristle-combs, segment X trapezoidal (Chick 2018, Aak et al. 2019, Molero-Baltaná et al. 2000; Fig. 1A-D).

### Lepisma saccharinum

Linnaeus, 1758

DE2DCDB9-AD96-5657-B5A3-492D42E50EBE

#### Materials

- **Type status:** Other material. **Occurrence:** recordedBy: Allan Selin; preparations: specimen in alcohol; **Taxon:** scientificName: Lepisma saccharinum; genus: Lepisma; specificEpithet: saccharinum; **Location:** country: Estonia; verbatimLocality: Maardu; decimalLatitude: 59.47111; decimalLongitude: 24.93972; coordinateUncertaintyInMeters: 50; **Identification:** identifiedBy: Kaarel Sammet; **Event:** eventDate: 18.XII.2003; **Record Level:** type: PhysicalObject; basisOfRecord: PreservedSpecimen
- **Type status:** Other material. **Occurrence:** recordedBy: Allan Selin; preparations: specimen in alcohol; **Taxon:** scientificName: Lepisma saccharinum; genus: Lepisma; specificEpithet: saccharinum; **Location:** country: Estonia; verbatimLocality: Maardu; decimalLatitude: 59.47111; decimalLongitude: 24.93972; coordinateUncertaintyInMeters: 50; **Identification:** identifiedBy: Kaarel Sammet; **Event:** eventDate: 18.XII.2003; **Record Level:** type: PhysicalObject; basisOfRecord: PreservedSpecimen
- **Type status:** Other material. **Occurrence:** recordedBy: Allan Selin; preparations: specimen in alcohol; **Taxon:** scientificName: Lepisma saccharinum; genus: Lepisma; specificEpithet: saccharinum; **Location:** country: Estonia; verbatimLocality: Maardu; decimalLatitude: 59.47111; decimalLongitude: 24.93972; coordinateUncertaintyInMeters: 50; **Identification:** identifiedBy: Kaarel Sammet; **Event:** eventDate: 15.I.2004; **Record Level:** type: PhysicalObject; basisOfRecord: PreservedSpecimen
- **Type status:** Other material. **Occurrence:** recordedBy: Tőnu Kesküla; preparations: pinned; **Taxon:** scientificName: Lepisma saccharinum; genus: Lepisma; specificEpithet: saccharinum; **Location:** country: Estonia; verbatimLocality: Kokora; decimalLatitude: 58.6333; decimalLongitude: 27.0002; coordinateUncertaintyInMeters: 500; **Identification:** identifiedBy: Kaarel Sammet; **Event:** eventDate: 16.V.2004; **Record Level:** type: PhysicalObject; basisOfRecord: PreservedSpecimen
- **Type status:** Other material. **Occurrence:** recordedBy: Tőnu Kesküla; preparations: pinned; **Taxon:** scientificName: Lepisma saccharinum; genus: Lepisma; specificEpithet: saccharinum; **Location:** country: Estonia; verbatimLocality: Tartu, Riia 181; decimalLatitude: 58.35667; decimalLongitude: 26.67861; coordinateUncertaintyInMeters: 50; **Identification:** identifiedBy: Kaarel Sammet; **Event:** eventDate: 17.II.2006; **Record Level:** type: PhysicalObject; basisOfRecord: PreservedSpecimen
- **Type status:** Other material. **Occurrence:** recordedBy: Tőnu Kesküla; preparations: pinned; **Taxon:** scientificName: Lepisma saccharinum; genus: Lepisma; specificEpithet: saccharinum; **Location:** country: Estonia; verbatimLocality: Tartu, Aardla 124; decimalLatitude: 58.35289; decimalLongitude: 26.68347; coordinateUncertaintyInMeters: 50; **Identification:** identifiedBy: Kaarel Sammet; **Event:** eventDate: 22.III.2006; **Record Level:** type: PhysicalObject; basisOfRecord: PreservedSpecimen
- **Type status:** Other material. **Occurrence:** recordedBy: Allan Selin; preparations: pinned; **Taxon:** scientificName: Lepisma saccharinum; genus: Lepisma; specificEpithet: saccharinum; **Location:** country: Estonia; verbatimLocality: Maardu; decimalLatitude: 59.471110; decimalLongitude: 24.93972; coordinateUncertaintyInMeters: 50; **Identification:** identifiedBy: Kaarel Sammet; **Event:** eventDate: 08.VI.2006; **Record Level:** type: PhysicalObject; basisOfRecord: PreservedSpecimen
- **Type status:** Other material. **Occurrence:** recordedBy: Allan Selin; preparations: pinned; **Taxon:** scientificName: Lepisma saccharinum; genus: Lepisma; specificEpithet: saccharinum; **Location:** country: Estonia; verbatimLocality: Maardu; decimalLatitude: 59.471110; decimalLongitude: 24.93972; coordinateUncertaintyInMeters: 50; **Identification:** identifiedBy: Kaarel Sammet; **Event:** eventDate: 15.I.2007; **Record Level:** type: PhysicalObject; basisOfRecord: PreservedSpecimen
- **Type status:** Other material. **Occurrence:** catalogNumber: IZBE0740006; recordedBy: Märt Kruus; preparations: specimen in alcohol; **Taxon:** scientificName: Lepisma saccharinum; genus: Lepisma; specificEpithet: saccharinum; **Location:** country: Estonia; verbatimLocality: Ignase; decimalLatitude: 58.25111; decimalLongitude: 26.83194; coordinateUncertaintyInMeters: 50; **Identification:** identifiedBy: Kaarel Sammet; **Event:** eventDate: 03.IX.2015; **Record Level:** type: PhysicalObject; basisOfRecord: PreservedSpecimen
- **Type status:** Other material. **Occurrence:** catalogNumber: IZBE0740007; recordedBy: Märt Kruus; preparations: specimen in alcohol; **Taxon:** scientificName: Lepisma saccharinum; genus: Lepisma; specificEpithet: saccharinum; **Location:** country: Estonia; verbatimLocality: Ignase; decimalLatitude: 58.25111; decimalLongitude: 26.83194; coordinateUncertaintyInMeters: 50; **Identification:** identifiedBy: Kaarel Sammet; **Event:** eventDate: 03.IX.2015; **Record Level:** type: PhysicalObject; basisOfRecord: PreservedSpecimen
- **Type status:** Other material. **Occurrence:** catalogNumber: IZBE0740000; recordedBy: Tőnu Kesküla; preparations: specimen in alcohol; **Taxon:** scientificName: Lepisma saccharinum; genus: Lepisma; specificEpithet: saccharinum; **Location:** country: Estonia; verbatimLocality: Tartu, Estonian University of Life Sciences; decimalLatitude: 58.39219; decimalLongitude: 26.69395; coordinateUncertaintyInMeters: 50; **Identification:** identifiedBy: Kaarel Sammet; **Event:** eventDate: 21.IX.2016; **Record Level:** type: PhysicalObject; basisOfRecord: PreservedSpecimen
- **Type status:** Other material. **Occurrence:** catalogNumber: IZBE0740002; recordedBy: Tőnu Kesküla; preparations: specimen in alcohol; **Taxon:** scientificName: Lepisma saccharinum; genus: Lepisma; specificEpithet: saccharinum; **Location:** country: Estonia; verbatimLocality: Tartu, Estonian University of Life Sciences; decimalLatitude: 58.39219; decimalLongitude: 26.69395; coordinateUncertaintyInMeters: 50; **Identification:** identifiedBy: Kaarel Sammet; **Event:** eventDate: 14.XII.2016; **Record Level:** type: PhysicalObject; basisOfRecord: PreservedSpecimen
- **Type status:** Other material. **Occurrence:** catalogNumber: IZBE0880018; recordedBy: Kaarel Sammet; preparations: specimen in alcohol; **Taxon:** scientificName: Lepisma saccharinum; genus: Lepisma; specificEpithet: saccharinum; **Location:** country: Estonia; verbatimLocality: Tartu, Emajőe 3; decimalLatitude: 58.38695; decimalLongitude: 26.71958; coordinateUncertaintyInMeters: 50; **Identification:** identifiedBy: Kaarel Sammet; **Event:** eventDate: 27.IV.2018; **Record Level:** type: PhysicalObject; basisOfRecord: PreservedSpecimen
- **Type status:** Other material. **Occurrence:** catalogNumber: IZBE0880020; recordedBy: Sirle Varusk; preparations: specimen in alcohol; **Taxon:** scientificName: Lepisma saccharinum; genus: Lepisma; specificEpithet: saccharinum; **Location:** country: Estonia; verbatimLocality: Tartu, Kreutzwaldi 52; decimalLatitude: 58.38758; decimalLongitude: 26.69523; coordinateUncertaintyInMeters: 50; **Identification:** identifiedBy: Kaarel Sammet; **Event:** eventDate: 25.V.2020; **Record Level:** type: PhysicalObject; basisOfRecord: PreservedSpecimen
- **Type status:** Other material. **Occurrence:** recordedBy: Allan Selin; preparations: specimen in alcohol; **Taxon:** scientificName: Lepisma saccharinum; genus: Lepisma; specificEpithet: saccharinum; **Location:** country: Estonia; verbatimLocality: Maardu; decimalLatitude: 59.471110; decimalLongitude: 24.93972; coordinateUncertaintyInMeters: 50; **Identification:** identifiedBy: Kaarel Sammet; **Event:** eventDate: 01.X.2020; **Record Level:** type: PhysicalObject; basisOfRecord: PreservedSpecimen
- **Type status:** Other material. **Occurrence:** catalogNumber: IZBE0880031; recordedBy: R. Lokk; preparations: specimen in alcohol; **Taxon:** scientificName: Lepisma saccharinum; genus: Lepisma; specificEpithet: saccharinum; **Location:** country: Estonia; verbatimLocality: Tartu, Kaunase pst. 36; decimalLatitude: 58.37239; decimalLongitude: 26.76815; coordinateUncertaintyInMeters: 50; **Identification:** identifiedBy: Kaarel Sammet; **Event:** eventDate: 06.XII.2020; **Record Level:** type: PhysicalObject; basisOfRecord: PreservedSpecimen

#### Notes

This species was recorded in Estonia as *Lepisma saccharina* by Remm (1966) and Vilbaste (1968). Widespread both in public buildings and private households.

### Thermobia domestica

(Packard, 1873)

D60BAB90-AFA2-58F8-83A8-F31FD4A8EBE8

#### Notes

*Thermobia domestica* was recorded from Estonia by Martin (2007). Its only confirmed occurrence was in a rural household in south-western Estonia in 2005. The species was identified, based on dead specimens and exuvia, but live specimens were also observed. The specimens were likely brought in with imported second-hand clothes. No voucher specimens have been preserved.

## Discussion

**Figure 2.**
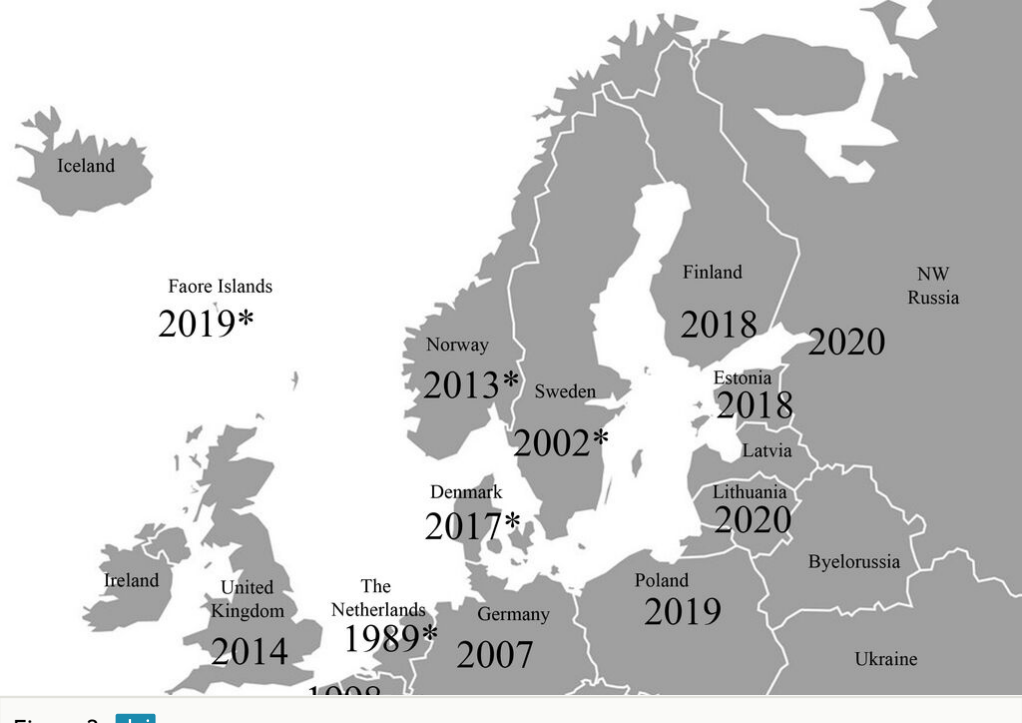
The years of first findings of *Ctenolepisma longicaudatum* from northern European countries and regions. The source references are: Belgium (Lock 2007), United Kingdom (Goddard et al. 2016), The Netherlands (Nierop and Hakbijl 2002, Schoelitsz and Brooks 2014), Germany (Meineke and Menge 2014), Denmark (Thomsen et al. 2019), Poland (Aak et al. 2019), Faroe Islands (Thomsen et al. 2019), Norway (Aak et al. 2019), Sweden (Pape and Wahlstedt 2002, Shah and Coulson 2020), Finland (Ueda 2020), NW Russia (Ueda 2020), Estonia (original data), Lithuania (Ueda 2020). The asterisk indicates a suspicion of an earlier observation (Schoelitsz and Brooks 2014, Thomsen et al. 2019, Anders Aak, pers. comm.).

The presence of *L. saccharinum* in Estonia has been well known for a long time. It is unclear when it first appeared in the Baltic Region, but it is notable that J. B. Fischer does not mention the species in his 1778 monograph on Livland’s fauna (c.f. Fischer 1778). The 'Handbook of Alien Species in Europe' lists *C. longicaudatum* as a cosmopolitan cryptogenic species (i.e. of unknown origin) (Drake 2009). In recent decades, it has been reported from several northern European countries and regions (Fig. 2). According to the public databases (GIBIF, Shah and Coulson 2020 and iNaturalist, Ueda 2020), the species has been recorded repeatedly from Helsinki, southern Finland since 2018, with first records from central Finland in 2020 and in the surroundings of Vilnius, Lithuania in 2019 (Ueda 2020). Moreover, there is an unconfirmed record from St. Petersburg, north-western Russia. However, there are no confirmed records from European Russia as yet (Vladimir Kaplin, pers. comm.) and also no records from Latvia (Voldemārs Spuņģis, pers. comm.). Some papers reporting new findings of the species in Europe hypothesise that the species was introduced considerably earlier, as it was already widely distributed in the country, for example, in Sweden (Pape and Wahlstedt 2002) and Faroe Islands (Thomsen et al. 2019). As for Estonia, it seems unlikely that the species has been overlooked for much longer, while the initial finding localities (Estonian University of Life Sciences Entomological collection and Estonian National Archive, both in Tartu) have been constantly monitored for potential pests. All current findings are from large public buildings, whereas there are, as yet, no records from private households (but is expected to be ultimately found in the latter). Compared with other synanthropic Zygentoma species, *C. longicaudatum* has much lower moisture demand and thus has a good chance of surviving in archives, libraries and museums, where there is plenty of suitable food for it (Aak et al. 2019). The species, expanding its range northwards (see Fig. 2), is considered a substantial pest especially of paper (see Fig. 1E,F, Szpryngiel 2018, Kulma et al. 2018). Therefore, its monitoring and being included to IPM (Integrated Pest Management) plans of museums, libraries and archives is inevitable (Querner 2015). Extended information on efficient and safe control can be found, for example, in Aak et al. 2020a, Aak et al. 2020b, Gutsmann 2019. There are currently no known established populations of *Thermobia domestica* in Estonia. However, the species is known for its requirement of higher temperature in order to successfully establish. Two other *Ctenolepisma* species, *C. lineatum* (Fabricius, 1775) and *C. calva* (Ritter, 1910) have been recently found in Norway (Hage et al. 2020), warranting further studies on Nordic Zygentoma diversity and distribution.

## Supplementary Material

XML Treatment for Ctenolepisma longicaudatum

XML Treatment for Lepisma saccharinum

XML Treatment for Thermobia domestica
